# Interferon-gamma release assay positivity in populations at high risk of TB infection

**DOI:** 10.5588/ijtldopen.25.0684

**Published:** 2026-04-13

**Authors:** A.F. Dagnew, L.L. Han, A. Cinar, D. Gaikwad, A.L. Garcia-Basteiro, M.T. Gler, S.R. Hadinegoro, W.A. Hanekom, J.R. Lama, M. Muyoyeta, S. Musala, V. Nduba, V.C. Rolla, T. Roy, J.S. Sutherland, S. Viegas, A. Wajja, T.M. Walker, R. Noble, L. Schlehuber, J. Sunshine, A.C. Schmidt, on behalf of the TBV02-E01 Study Group

**Affiliations:** 1 Gates Medical Research Institute, Clinical Development, Cambridge, MA, USA;; 2 Department of Pulmonary Medicine, PCMC’S PGI YCM Hospital, Pune, India;; 3 Centro de Investigação em Saúde de Manhiça (CISM), Maputo, Mozambique;; 4 Barcelona Institute for Global Health, ISGlobal, Barcelona, Spain;; 5 Centro de Investigación Biomédica en Red de Enfermedades Infecciosas (CIBERINFEC), Barcelona, Spain;; 6 TB HIV Innovations and Clinical Research Foundation, Cavite, Philippines;; 7 Department of Child Health, Faculty of Medicine, University of Indonesia, Jakarta, Indonesia;; 8 Africa Health Research Institute, KwaZulu-Natal, South Africa;; 9 Asociación Civil Impacta Salud y Educación, Lima, Peru;; 10 Centre for Infectious Disease Research in Zambia (CIDRZ), Lusaka, Zambia;; 11 National Tuberculosis Program/National TB Reference Laboratory, Kinshasa City, Democratic Republic of the Congo;; 12 Kenya Medical Research Institute, Centre for Respiratory Diseases Research (CRDR), Nairobi, Kenya;; 13 National Institute of Infectious Diseases Evandro Chagas - Fiocruz, Rio de Janeiro, Brazil;; 14 IRD Global, Bangladesh Country Office, Dhaka, Bangladesh;; 15 Vaccines and Immunity Theme, Medical Research Council Unit the Gambia at the London School of Hygiene and Tropical Medicine, Fajara, the Gambia;; 16 Instituto Nacional de Saúde, Marracuene, Mozambique;; 17 Medical Research Council/Uganda Virus Research Institute and London School of Hygiene and Tropical Medicine Uganda Research Unit, Entebbe, Uganda;; 18 Department of Clinical Research, London School of Hygiene and Tropical Medicine, London, UK;; 19 Oxford University Clinical Research Unit, Ho Chi Minh City, Vietnam;; 20 Centre for Tropical Medicine and Global Health, Nuffield Department of Medicine, University of Oxford, Oxford, UK.

**Keywords:** tuberculosis, TBI, epidemiology, IGRA, vaccine efficacy trial

## Abstract

**BACKGROUND:**

Phase 3 TB vaccine trials are challenging in low-incidence settings due to the need for large sample sizes and extended follow-up. This global, observational study evaluated population-based interferon-gamma release assay (IGRA) status – a measure of TB infection (TBI), as a proxy for TB incidence to identify trial sites in high-incidence areas.

**METHODS:**

Participants (15–34 years) were recruited from 45 sites in 14 countries. IGRA status at Day 1 and Month 12, association of IGRA status with age, and IGRA conversion (TBI) were assessed.

**RESULTS:**

Among 7,164 enrolled participants, Day 1 IGRA positivity varied across sites and within countries, with the highest prevalence observed in South Africa (58.7%, site 1006; 53.1%, site 1010; 51.9%, site 1007) and the Democratic Republic of Congo (50.0%, site 2303). IGRA positivity was generally higher among older participants. At Month 12, sites with highest IGRA conversion were observed in the Philippines (32.3%, site 1507) and Zambia (30.6%, site 1303).

**CONCLUSION:**

In TB vaccine efficacy trials with clinical endpoints, selecting sites with the highest TB incidence is critical to optimise sample size and follow-up duration. Site-level IGRA status could inform site selection by identifying communities at increased risk of *Mycobacterium tuberculosis* infection.

TB is a leading cause of death due to a single infectious agent, *Mycobacterium tuberculosis* (*Mtb*), with an estimated 1.25 million deaths worldwide in 2023.^1^ The burden of pulmonary TB is highest among adolescents and adults, who are the primary source of *Mtb* transmission, and modelling suggests that a vaccine aiming to prevent pulmonary TB in these groups could be cost-effective even at relatively low efficacy levels.^2–4^ BCG (Bacillus Calmette-Guérin) is currently the only TB vaccine, but its protection starts to decline in adolescence and it offers minimal or no protection against pulmonary TB in adults.^5^ New vaccines are urgently needed to help end the TB epidemic. Within resource-limited countries, where TB is most prevalent, the incidence rate of the disease could vary between neighbourhoods.^6^ As such, accurate site-specific epidemiology data are needed to support site selection for TB vaccine efficacy trials. It is critical to conduct efficacy trials in communities with the highest possible TB incidence to optimise both sample size and follow-up duration. Given the large sample size needed to measure site-level TB incidence, we used interferon-gamma release assay (IGRA) status as a proxy for TB infection.

The primary purpose of this observational study was to evaluate IGRA status among people living in settings of high TB risk to identify sites with suitable local epidemiology in anticipation of a planned phase 3 TB vaccine efficacy trial (NCT06062238). This study also intended to build site capacity and clinical research experience, helping sites become familiar with procedures expected in future TB vaccine trials.

## METHODS

This was a global, observational epidemiologic study (NCT05190146) designed to assess IGRA positivity at the site level. To evaluate TB signs and symptoms, participants visited the study site every 6 months after the Day 1 screening visit. IGRA status was assessed on Day 1 and Month 12. Eligible participants (15–34 years inclusive) were capable of giving informed consent or informed assent and were considered to be at high risk for *Mtb* infection and progression to TB disease. High risk was defined as living in a community with a high burden of TB. Exclusion criteria included prior experimental *Mtb* vaccination, active TB within the last 24 months, or uncontrolled chronic conditions.

Across sites, teams were instructed to recruit participants who resided in communities classified locally as having a high TB burden. However, implementation of this instruction was not completely uniform. At most sites, recruiters specifically targeted communities known or believed to have a high TB burden. The recruitment strategy focused on the broad general population within the local community, rather than specifically targeting groups such as household contacts or health care workers.

### Procedures

On Day 1, assessments included medical history review, demographics, and full physical examination. Blood samples were drawn for IGRA (QuantiFERON®-TB Gold Plus [QFT-Plus] assay; Qiagen, MD, USA) and HIV testing (Supplementary Data Table S1). Positive, negative, and indeterminate IGRA status were determined based on manufacturer’s instructions.^7^ Active surveillance for signs and symptoms compatible with incident TB disease continued throughout the study. Suspected TB cases (defined as a participant presenting with unexplained cough, unexplained fever, night sweats, unintentional weight loss, or haemoptysis) prompted unscheduled visits for medical evaluation and collection of sputum and blood samples. Sputum samples were collected at three different suspected TB visits, within a maximum period of 1 week. A laboratory-confirmed pulmonary TB case was defined as a participant with suspected pulmonary TB who had at least one positive *Mtb* culture or at least one positive GeneXpert® MTB/RIF Ultra (Xpert Ultra; Cepheid USA) test result, based on the three sputum samples collected. Trace results from Xpert Ultra assay were considered positive. Clinical TB is defined in the Supplementary Data. Samples for HIV and IGRA testing were collected at one of the three suspected TB visits. Samples were also collected for IGRA testing at Month 12. Sample processing and testing at all sites is described in the Supplementary Data.

### Outcomes

The primary objective was the prevalence of IGRA positivity by site. IGRA status at Day 1 was summarised by site and by country.

Secondary objectives were the association of age with IGRA positivity, by site, and overall incidence rate of suspected and laboratory-confirmed pulmonary TB. IGRA positivity was summarised by age groups (15–24 and 25–34 years) for each site and country. Incidence rates of suspected, laboratory-confirmed, and clinical TB were summarised by country. Exploratory endpoints compared IGRA status at Month 12 versus Day 1.

### Statistical analysis

The all-enrolled analysis set comprised all screened participants who fulfilled eligibility requirements. The per-protocol set included participants who met all the inclusion and none of the exclusion criteria and had IGRA status available at Day 1, including participants with suspected TB. The per-protocol was used for analysing all study objectives. This was an estimation study, and no formal statistical hypotheses were tested. The percentage of participants with positive IGRA was calculated as all participants with positive IGRA status divided by the total number of participants who had an IGRA test result (positive/negative/indeterminate) available at Day 1. Two-sided 95% confidence intervals (CIs) were based on the binomial distribution and were calculated using the Wald or Clopper–Pearson method, as appropriate. The Wald method, including correction for continuity, was used for sample sizes of ≥100.

A multilevel mixed-effects logistic regression model was used to assess the association between age and the odds of a positive IGRA at screening. The model included a random effect for site, nested within country. Covariates for baseline characteristics including age (continuous), sex (male/female), body mass index (BMI; continuous), BCG scar (Yes/No), prior or baseline TB disease prevention treatment (TPT; Yes/No), Hispanic or Latino ethnicity (Yes/No), race (six categories), health care profession (Yes/No), and positive HIV status (Yes/No) were considered for the model at 0.1 significance level. Younger age (15–24 years) was used as the reference group, and therefore higher odds ratios (ORs) were indicative of higher odds of IGRA positivity for older ages. ORs >1 illustrate the increase in odds for each 1-year increase in age. Incidence rates of suspected and laboratory-confirmed pulmonary TB were calculated as described in the Supplementary Data.

### Ethical statement

The study was conducted in compliance with the Good Clinical Practice guidelines per the International Council for Harmonisation E6(R2), and all applicable local and national regulatory requirements. The protocol was approved by the relevant independent ethics committees.

## RESULTS

This study was conducted from 20 December 2021 (first participant enrolled) through 16 August 2024 (last participant last visit), across 45 sites in 14 countries (Table 1; Supplementary Data Table S2). Of 7,280 participants screened, 7,164 (98.4%) were enrolled and 7,135 (98.0%) were included in the per-protocol set (Supplementary Data Figure S1). Demographics and baseline characteristics by sites are described in Supplementary Data Table S3. The median (interquartile range [IQR]) follow-up duration for all enrolled participants was 17.1 months (16.2–9.3). The site with the shortest duration in the study was site 2201 in Indonesia (median [IQR] of 10.9 months [10.64–11.10]), and with the longest duration was site 1008 in South Africa (median [IQR] of 21.9 months [21.85–22.67]).

**Table 1.** Demographics and baseline characteristics by country (per-protocol set).

Characteristics	Bangladesh (N = 160)	Brazil (N = 319)	Democratic Republic of Congo (N = 471)	The Gambia (N = 159)	India (N = 160)	Indonesia (N = 480)	Kenya (N = 639)
Mean age, years (SD)	25.31 (4.67)	25.13 (4.19)	23.49 (5.12)	23.01 (5.71)	25.41 (4.87)	23.58 (4.62)	25.19 (4.54)
15–24 years	75 (46.9)	147 (46.1)	291 (61.8)	95 (59.7)	71 (44.4)	299 (62.3)	302 (47.3)
25–34 years	85 (53.1)	172 (53.9)	180 (38.2)	64 (40.3)	89 (55.6)	181 (37.7)	337 (52.7)
Females	68 (42.5)	204 (64.0)	193 (41.0)	109 (68.6)	50 (31.3)	201 (41.9)	342 (53.5)
Race							
Black	0	117 (36.7)	471 (100)	159 (100)	0	0	639 (100)
White	0	148 (46.4)	0	0	0	0	0
Asian	156 (97.5)	0	0	0	160 (100)	480 (100)	0
Southern American Indian	3 (1.9)	0	0	0	0	0	0
Southern African coloured	1 (0.6)	0	0	0	0	0	0
Other	0	1 (0.3)	0	0	0	0	0
Mixed		53 (16.6)					
Ethnicity							
Not Hispanic or Latino	160 (100)	160 (50.2)	471 (100)	159 (100)	159 (99.4)	480 (100)	637 (99.7)
Hispanic or Latino	0	159 (49.8)	0	0	1 (0.6)	0	2 (0.3)
Health care profession (student or worker)	0	49 (15.4)	35 (7.4)	1 (0.6)	0	6 (1.3)	10 (1.6)
Mean BMI, kg/m^2^ (SD)	22.60 (4.19)	27.18 (6.55)	21.35 (3.36)	21.75 (4.32)	22.84 (2.36)	23.69 (6.00)	23.61 (5.15)
Underweight: <18.5 kg/m^2^	24 (15.0)	10 (3.1)	77 (16.3)	34 (21.4)	1 (0.6)	91 (19.0)	48 (7.5)
Obese: ≥30 kg/m^2^	9 (5.6)	86 (27.0)	9 (1.9)	10 (6.3)	0	73 (15.2)	81 (12.7)
BCG scar present	158 (98.8)	306 (95.9)	420 (89.2)	103 (64.8)	159 (99.4)	391 (81.5)	599 (93.7)
Positive HIV status	0	8 (2.5)	0	3 (1.9)	1 (0.6)	1 (0.2)	40 (6.3)
Prior or baseline TPT	0	0	0	0	0	0	0

Characteristics	Mozambique (N = 319)	Peru (N = 316)	Philippines (N = 1,117)	South Africa (N = 1,410)	Uganda (N = 320)	Vietnam (N = 626)	Zambia (N = 639)
Mean age, years (SD)	22.99 (5.07)	25.00 (4.57)	24.52 (4.92)	24.58 (4.87)	24.45 (4.34)	23.65 (4.85)	23.32 (4.75)
15–24 years	228 (71.5)	160 (50.6)	604 (54.1)	726 (51.5)	173 (54.1)	387 (61.8)	386 (60.4)
25–34 years	91 (28.5)	156 (49.4)	513 (45.9)	684 (48.5)	147 (45.9)	239 (38.2)	253 (39.6)
Females	158 (49.5)	188 (59.5)	551 (49.3)	766 (54.3)	161 (50.3)	322 (51.4)	418 (65.4)
Race							
Black	319 (100)	0	0	1,254 (88.9)	320 (100)	0	639 (100)
White	0	2 (0.6)	0	13 (0.9)	0	0	0
Asian	0	0	1,117 (100)	0	0	626 (100)	0
Southern American Indian	0	5 (1.6)	0	0	0	0	0
Southern African coloured	0	0	0	142 (10.1)	0	0	0
Other	0	1 (0.3)	0	0	0	0	0
Mixed	0	308 (97.5)	0	1 (0.1)	0	0	0
Ethnicity							
Not Hispanic or Latino	317 (99.4)	0	1,116 (99.9)	1,397 (99.1)	319 (99.7)	626 (100)	639 (100)
Hispanic or Latino	2 (0.6)	316 (100)	1 (0.1)	13 (0.9)	1 (0.3)	0	0
Health care profession (student or worker)	1 (0.3)	12 (3.8)	18 (1.6)	10 (0.7)	9 (2.8)	78 (12.5)	20 (3.1)
Mean BMI, kg/m^2^ (SD)	22.33 (3.86)	27.42 (5.57)	24.63 (5.05)	24.45 (6.95)	23.17 (4.50)	22.59 (4.44)	22.04 (4.21)
Underweight: <18.5 kg/m^2^	30 (9.4)	5 (1.6)	93 (8.3)	244 (17.3)	26 (8.1)	99 (15.8)	86 (13.5)
Obese: ≥30 kg/m^2^	16 (5.0)	84 (26.6)	156 (14.0)	280 (19.9)	27 (8.4)	50 (8.0)	36 (5.6)
BCG vaccine scar present	308 (96.6)	249 (78.8)	869 (77.8)	793 (56.2)	242 (75.6)	564 (90.1)	426 (66.7)
Positive HIV status	29 (9.1)	3 (0.9)	6 (0.5)	154 (10.9)	20 (6.3)	5 (0.8)	66 (10.3)
Prior or baseline TPT	0	2 (0.6)	6 (0.5)	13 (0.9)	0	1 (0.2)	0

Data are n (%) unless otherwise specified. Denominator for percentages based on the number of participants in the per-protocol set within each country.

BCG = Bacillus Calmette-Guerin; BMI = body mass index; TPT = TB prevention treatment.

### IGRA positivity

**Figure 1.**
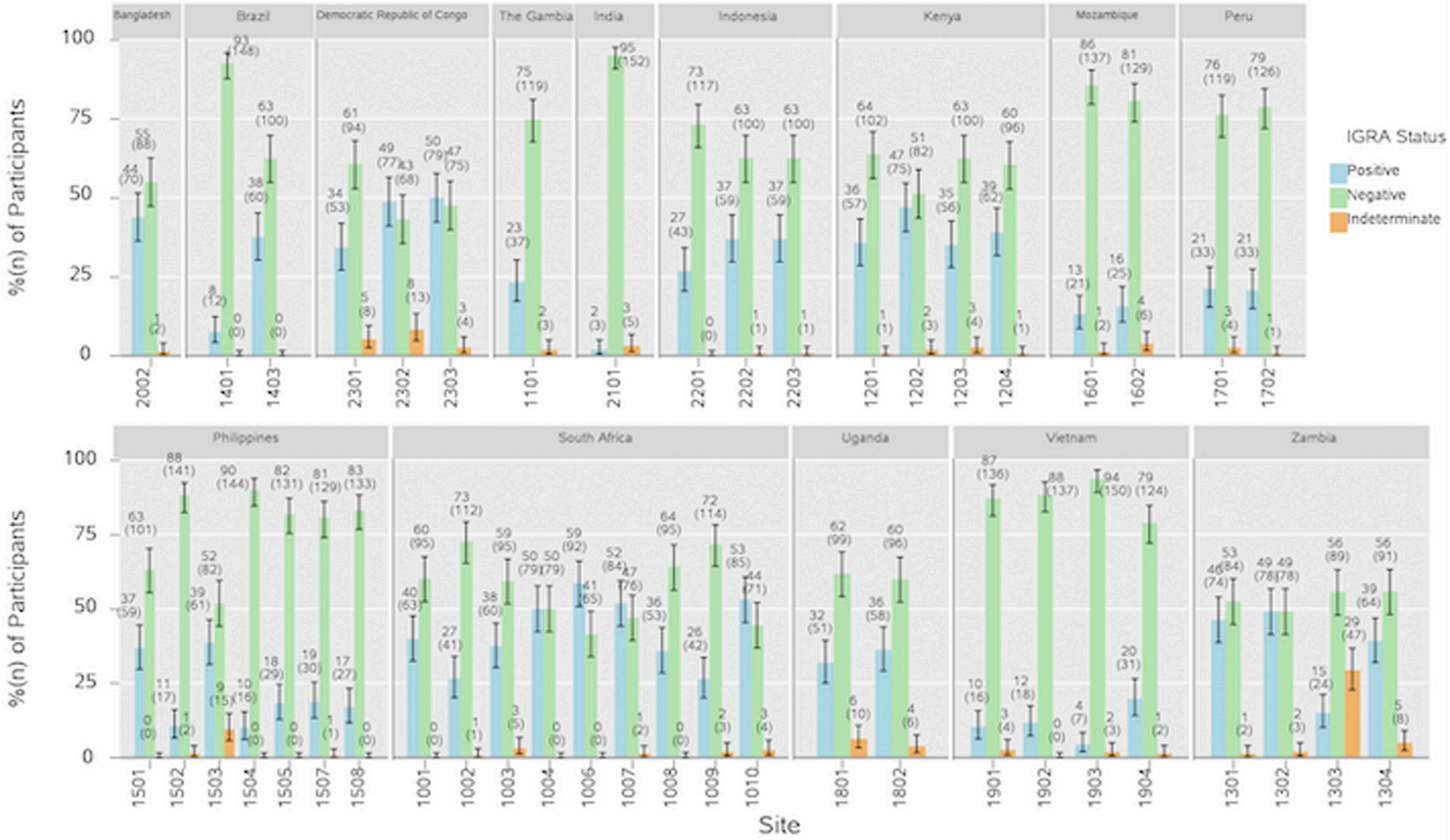
IGRA status at Day 1, by site (per-protocol set). Figure shows participants with IGRA status at Day 1, grouped by sites at each country. Each bar represents the percentage of participants with IGRA positive (blue), negative (green), or indeterminate (orange) status. Error bars represent 95% CIs. IGRA = interferon-gamma release assay; CI = confidence interval.

**Figure 2.**
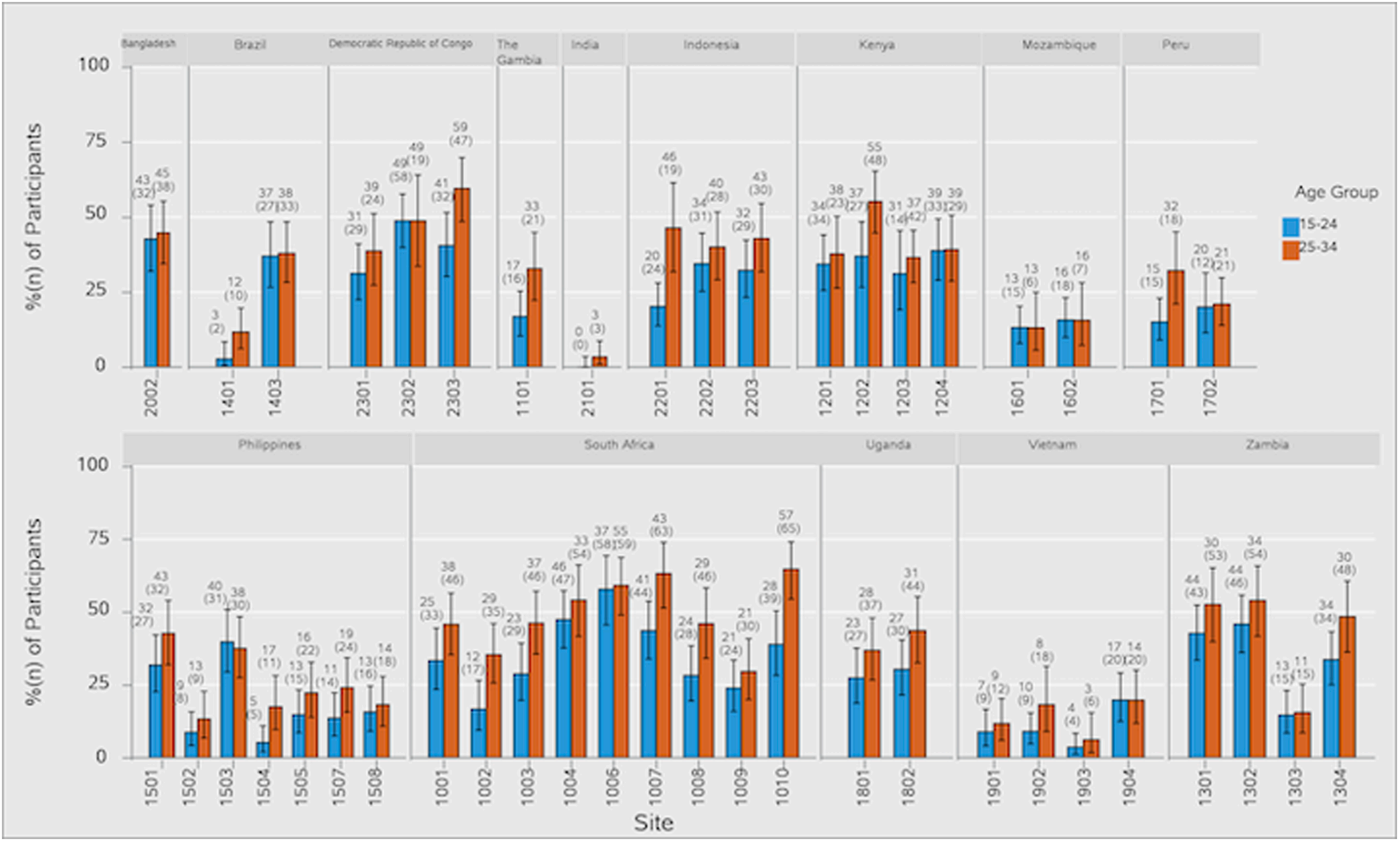
IGRA positivity at Day 1 by age category and site (per-protocol set). Figure shows participants with IGRA-positive status at Day 1 stratified by age categories and grouped by sites at each country. Each pair of bar represents the percentage of IGRA-positive participants in 15–24 years (blue) and 25–34 years (orange) age category. Error bars represent 95% CIs. IGRA = interferon-gamma release assay; CI = confidence interval.

IGRA positivity at Day 1 varied across sites, both within and between countries (Table 2). The sites with the highest Day 1 IGRA positivity were in South Africa (58.7%, site 1006; 53.1%, site 1010; 51.9%, site 1007; 50.0%, site 1004) and the Democratic Republic of Congo (DRC; 50.0%, site 2303) (Figure 1; Supplementary Data Table S4). At most of the sites, the percentage of participants with positive IGRA was greater among those aged 25–34 years compared with 15–24 years (Figure 2; Supplementary Data Table S5). Among participants aged 15–24 years, IGRA positivity was highest in South Africa, followed by the DRC. In the 25–34 years age group, IGRA positivity of ≥50% was observed at multiple sites including in South Africa, the DRC, Kenya, and Zambia.

**Table 2.** Incidence rate of laboratory-confirmed pulmonary TB by country (per-protocol set).

Country	Number of participants in per-protocol set	Number of participants with first laboratory-confirmed pulmonary TB	Person years	Incidence rate (IR/100, 000)	95% CI for IR
The Gambia	159	1	255.5	391.4	9.9–2,149.8
India	160	1	162.4	615.7	15.6–3,382.1
Indonesia	480	2	439.9	454.6	55.1–1,633.4
Kenya	639	3	973.7	308.1	63.6–896.4
Peru	316	1	449.1	222.7	5.6–1,231.8
Philippines	1,117	4	1,708.0	234.2	63.9–597.9
South Africa	1,410	9	2327.8	386.6	177.0–731.9
Vietnam	626	2	896.9	223.0	27.0–802.1
Zambia	639	1	898.8	111.3	2.8–617.7

Bangladesh, Brazil, Democratic Republic of Congo, Mozambique, and Uganda had no cases of laboratory-confirmed pulmonary TB. The laboratory-confirmed pulmonary TB incidence rates were calculated as the number of first laboratory-confirmed pulmonary TB event presented during the study follow-up period divided by the number of participant days at risk during the follow-up period and expressed as the number of events per 100,000 participant-years. The corresponding 95% two-sided confidence interval associated with the incidence rate of laboratory-confirmed pulmonary TB overall was derived based on binomial distribution assumption.

CI = confidence interval; IR = incidence rate.

### Association of age with IGRA status

**Figure 3.**
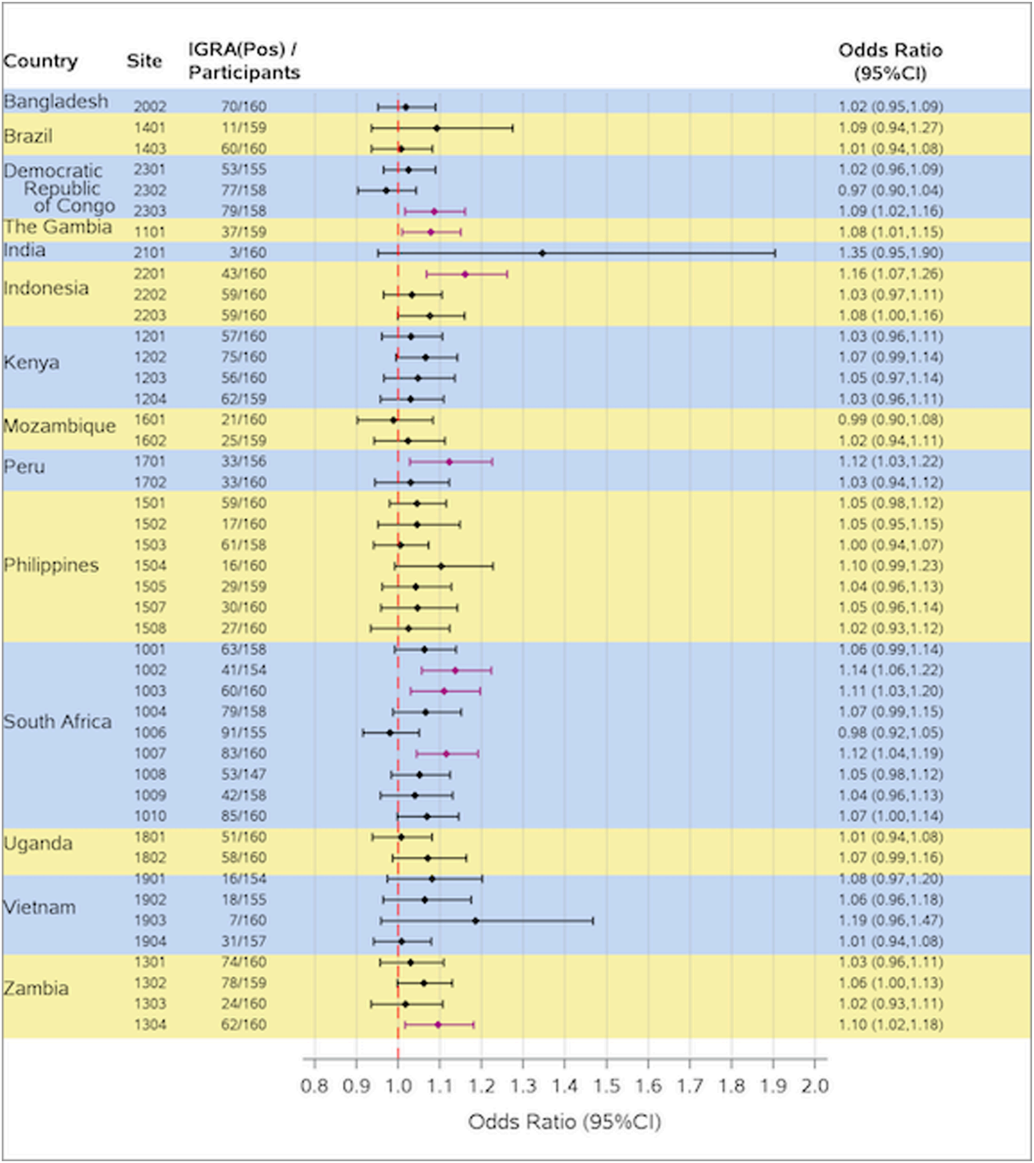
Association of age with the odds of a positive IGRA status at Day 1 (per-protocol set). Odds ratios (ORs) were based on a multilevel mixed-effects logistic regression model including a random effect for site (nested within country) and covariates for baseline characteristics including age (continuous), sex, BMI, prior or baseline TPT, and BCG scar. Younger age was used as the reference group. Horizontal purple lines indicate a greater than 1 lower bound of OR estimates. BCG = Bacillus Calmette-Guérin; BMI = body mass index; CI = confidence interval; IGRA = interferon-gamma release assay; TPT = TB preventive treatment.

The multilevel mixed-effect regression model found older age, males, absence of BCG scar, having prior or baseline TPT, and higher baseline BMI to be positively associated with having a positive IGRA result at screening. Among the 45 sites, nine had lower bounds of the 95% CI for the adjusted OR exceeding 1 for the association between age and IGRA positivity at Day 1 (Figure 3). For these nine sites, the odds of a positive IGRA status at Day 1 increased for each 1-year increase in age, with the highest estimated OR of 1.16 in Indonesia (site 2201). For the remaining 36 sites, 95% CIs for ORs encompassed 1 indicating no statistical significance. Ethnicity, race, health care profession, and HIV status were found to be not statistically significant and therefore were not included in the final model.

### IGRA conversions and reversions

At Month 12, IGRA conversion (IGRA negative at Day 1 and positive at Month 12) greater than 10% was observed at 11 sites: one site in Bangladesh, two in the DRC, two in Indonesia, four in the Philippines, one in Vietnam, and one in Zambia (Supplementary Data Table S6). Among these, site 1303 in Zambia and site 1507 in the Philippines reported 30.6% and 32.3% conversion, respectively, and the remaining nine sites reported conversion ranging from 10.5% in Indonesia to 18.4% in Philippines. All other sites reported IGRA conversion below 10%, with the lowest percentage observed in India (1.5%). IGRA reversion at Month 12 (IGRA positive at Day 1 and negative at Month 12) of greater than 10% was observed at two sites: site 1201 in Kenya (11.9%) and site 1503 in the Philippines (10.7%; Supplementary Data Table S6).

### Incidence rates

The incidence rate for suspected pulmonary TB was highest in South Africa (12,441.7/100,000), followed by Brazil (4,596.6/100,000) and Uganda (3,745.8/100,00; Supplementary Data Table S7). A total of 24 cases of laboratory-confirmed pulmonary TB were identified in nine countries, with incidence rates ranging from 111.3/100,000 in Zambia to 615.7/100,000 in India (Table 2; Supplementary Data Table S8). Additionally, 31 clinical TB cases were reported in seven countries: Brazil, Indonesia, Kenya, the Philippines, South Africa, Vietnam, and Zambia (Supplementary Data Table S9).

## DISCUSSION

Accessing reliable TB incidence data remains a challenge in areas of high TB risk.^8^ This global observational study evaluated 45 sites in 14 countries to inform site selection and feasibility for a phase 3 TB vaccine efficacy trial, by evaluating IGRA positivity at Day 1 as a proxy for TB incidence to identify trial sites in high-incidence areas. In addition, the study evaluated TB incidence over a median duration of 17.1 months, providing insights into IGRA conversion and reversions and promoting familiarity with operational processes relevant to future phase 3 trials.

Overall, we observed substantial variability in IGRA positivity at Day 1 across sites both within and between countries. This underscores the importance of strategically selecting sites to enrich trial populations with individuals at highest risk for TB, thereby optimising the likelihood of observing clinical endpoints in an event-triggered phase 3 vaccine efficacy trial. The IGRA positivity percentage was generally higher among older participants, with higher odds of a positive IGRA status among older participants, indicating cumulative risk of exposure to *Mtb* over time.^9^

Across study sites, IGRA conversion percentages varied widely, ranging from 1.5% in India to 32.3% in Philippines, highlighting the heterogeneity in TB transmission dynamics across high-burden regions. Notably, all nine sites in South Africa observed relatively low conversion, ranging from 2.8% to 5.3% over 1 year. These IGRA conversion rates are lower than the average conversion rate of 8% per person-year observed in a recent trial in 10- to 18-year-old adolescents in South Africa, but similar to conversion rates observed outside the Western Cape Province.^10^ The low IGRA conversion percentage, combined with the high percentage of baseline IGRA positivity observed at South African sites, suggests that *Mtb* infection occurs at a younger age in the communities enrolled in South Africa. This finding underscores the importance of understanding local transmission dynamics and identifying the appropriate age groups to target for a phase 3 TB vaccine trial with a clinical endpoint (for example, laboratory-confirmed pulmonary TB).

The highest IGRA conversion occurred at one site in Philippines (32.3%) and one site in Zambia (30.6%). We hypothesise that this high conversion could be due to health care disruptions during the COVID-19 pandemic, which may have led to delayed detection and treatment of TB patients in these communities, resulting in increased transmission. IGRA reversion varied from 0 to 11.9%, which is lower than that reported in a recent meta-analysis (22.8%),^11^ and often lower than IGRA conversion percentages across the different sites over the 17.1 months of follow-up duration in the current study. IGRA reversion percentages are difficult to interpret when limited to use of only two timepoints, as distinguishing between transient and sustained reversion is improved with sequential measurements over time.^12,13^

Although the QFT-Plus assay uses interferon-gamma levels ≥0.35 IU/mL in its positivity threshold, results near this cut-off are prone to both biological and technical variability.^12^ As such, some of the observed conversions and reversions may not reflect a true change in IGRA status, due to values being close to the diagnostic threshold and in the ‘uncertainty zone’. Nevertheless, these findings contribute a broader understanding of IGRA conversion and reversion patterns in general community settings, providing a useful reference point for hypothesis generation in future studies on TB vaccines.

The case definition for laboratory-confirmed TB required that at least one sputum sample collected from participants with suspected TB had to be positive by culture and/or Xpert Ultra. Among the cases detected, the majority (15/24) were positive by both culture and Xpert Ultra and/or had multiple sputum samples positive. Interestingly, nine participants had one positive TB result, of which eight were IGRA negative and one was positive at Day 1. Six of eight participants remained IGRA negative at the suspected TB visit and five of eight were not treated for TB. A more stringent case definition requiring multiple positive test results may be more reflective of true TB disease.

The main strength of this study is that, for many countries and sites, this is an opportunity to understand the epidemiology of IGRA status in a population-based setting. In addition, while IGRA conversions and reversions can vary due to biological and assay-related factors,^14^ this study provides valuable empirical data on IGRA conversion and reversion in community populations from high-TB-burden settings. All participants were evaluated for changes in IGRA status between Day 1 and Month 12, enabling estimates of both IGRA conversion and reversion percentages in community-based cohorts, rather than in populations limited to household contacts or health care workers, as is commonly reported in the literature.^11,15–17^ Another benefit of this operationally complex and challenging study is that it provides valuable lessons learned for sites and for sponsors, for use in optimising plans for future trials. Our study also has some limitations. The eligibility criteria did not clearly specify that participants with suspected pulmonary TB should be excluded from trial participation. However, we only identified one participant (site 1002, South Africa) with suspected TB at Day 1 who was later confirmed to have had laboratory-confirmed TB. Because high-risk communities were pragmatically identified and recruitment strategies varied between sites, differences in IGRA positivity across sites and countries observed in this study should be viewed as descriptive and hypothesis-generating rather than precise comparative estimates of underlying population risk. These findings are most appropriately used to inform recruitment strategies for the M72/AS01_E-4_ phase 3 trial and highlighting potential TB ‘hotspot’ areas within participating sites, rather than drawing firm conclusions about differences between countries or sites. Given the pragmatic sampling strategy and the primary goal of identifying high-risk communities for the M72/AS01_E-4_ phase 3 trial, the results of this study are not representative of any pre-specified national or subnational population and may not be generalisable beyond the communities from which participants were recruited.

## CONCLUSION

This large-scale global epidemiologic study evaluated population-based IGRA positivity to identify communities at increased risk of *Mtb* infection using IGRA as a proxy for TB incidence. For sites participating in phase 3 TB vaccine efficacy trials with clinical endpoint, it is essential that these trials be conducted in communities with the highest possible TB incidence rates to optimise sample size and follow-up duration and to enable efficient enrolment – particularly when the target population is IGRA positive.

## Supplementary Material
