# Supplementary material for: Interferon-gamma release assay positivity in populations at high risk of TB infection
Source: IJTLD Open. 2026 Apr 13;3(4):232–40. doi: 10.5588/ijtldopen.25.0684 (PMC13080303; doi:10.5588/ijtldopen.25.0684)

Supplementary Appendix

Supplement to: Dagnew AF, Han LL, Cinar A et al. Interferon-gamma release assay positivity in populations with high risk of *Mycobacterium tuberculosis* infection.

Methods

Study Design

Remote contact visits were conducted every 2 months, and unscheduled in-person visits were arranged if TB was suspected during these contacts.

During the study (maximum duration 30 months), sites were evaluated for participation in the investigational M72/AS01$_{E-4}$ phase 3 clinical trial (NCT06062238). Sites selected for the phase 3 trial were terminated from this epidemiologic study around the time of phase 3 trial initiation. Sites not selected to participate in the phase 3 trial were terminated after all participants had completed their Month 12 assessment. Whenever possible, a discontinuation visit was scheduled for participants who discontinued or withdrew from the study.

Participants

Additional inclusion criteria included agreement to stay in contact with the site for the duration of the study with no current plans to relocate from the study area for the duration of the study and providing updated contact information as necessary.

Procedures

Full physical examination comprised examination of BCG scar, assessments of the cardiovascular, respiratory, gastrointestinal and neurological systems, and assessment of height and weight.

Suspected TB cases identified during the screening visit (Visit 1), or at any point throughout the study, were referred for further evaluation and treatment according to the local standard of care. A clinical TB case was defined as a participant for whom a clinician diagnosed TB or initiated TB treatment, in the absence of laboratory-confirmed pulmonary TB as conducted at a sponsor laboratory for the study.

For IGRA samples, Bio Analytical Research Corporation (BARC) handled processing and testing for all South African sites. All other sites did their own processing, with testing at Vimta Laboratories Limited (India) or BARC (all other countries). The sputum samples from all countries, except India, were processed and tested at BARC. For samples from India, processing and testing was done at Vimta Laboratories Limited. For HIV testing, sites in all countries, except India, used point-of-care kits (**Table S1**) if available, otherwise ELISA was done at BARC. In India, due to unavailability of these kits, only ELISA was used with processing and testing handled by Vimta Laboratories Limited. The details of assays conducted are presented in **Table S1.** Blood samples collected for exploratory biomarkers analyses are described elsewhere (Sunshine J *et al.*, under review).

An interim medical history was evaluated at 6-month visits, followed by a focused physical examination if indicated by the medical history. After Day 1, anti-TB drugs were also recorded.

Statistical Analyses

Incidence rates of suspected TB or laboratory-confirmed pulmonary TB were calculated as the number of events presented during the study follow-up period, divided by the number of participant days at risk during the follow-up period, which was calculated as the number of days from Day 1 until the date of the event for participants who experienced the event, and the number of days during the follow-up period (to the earliest of death, loss to follow-up [date of last contact] or end-of-study/discontinuation visit) for participants who did not present with the event. The 95% CIs for these incidence rates were provided based on the normal approximation to the binomial distribution.

Table S1: Assays and their manufacturers

Assays information		Manufacturer
Assay	Specimen	
**HIV Confirmatory Tests**		
Determine HIV 1/2	Serum	Alere (USA)

OraQuick HIV Test	Serum	OraSure Technologies (USA)
**Interferon-gamma release assay (IGRA)**		
QuantiFERON®-TB Gold Plus	Blood	Qiagen (USA)
**Direct Mycobacterial Detection Tests**		
Mycobacterial Growth Indicator Tube test - MGIT™ (liquid media)	Sputum	BACTEC (USA)
Xpert® MTB/RIF Ultra	Sputum	Cepheid (USA)

Results

Participants' characteristics and disposition

Early discontinuation was highest in the Democratic Republic of Congo (DRC; 84/478 [17.6%]) and lowest in India (0/160 [0%]), with most common reasons being participant withdrawal and loss to follow-up (**Figure S1, Table S2**).

Female participants ranged from 31.3% (50/160) in India to 68.6% (109/159) in the Gambia (**Table S3**). The mean age was largely similar across countries (range 22 to 25 years), with generally a higher percentage of 15–24 years old participants than 25–34 years old, except in Bangladesh, Brazil, India and Kenya. Participants aged 15–24 years ranged between 44.4% (71/160) in India to 71.5% (228/319) in Mozambique.

At baseline, the percentage of people living with HIV (PLHIV) was highest in South Africa (10.9% [154/1410]), Zambia (10.3% [66/639]), and Mozambique (9.1% [29/319]) (**Table 1**). Bangladesh and the DRC had no positive HIV participants at baseline. Presence of BCG scars was lowest in South Africa (56.2% [793/1410]), the Gambia (64.8% [103/159]) and Zambia (66.7% [426/639]). TB disease prevention treatment (TPT) received prior to study enrolment or at baseline was reported in <1% of participants overall (**Table 1**).

TB disease

None of the participants with clinical TB were identified as laboratory-confirmed TB cases. One participant in South Africa had suspected TB at screening and was subsequently diagnosed with laboratory-confirmed TB. In Zambia, three participants with suspected TB at screening were later classified as clinical TB cases.

IGRA and HIV status of participants with laboratory-confirmed pulmonary TB

Among the participants with laboratory-confirmed pulmonary TB, the majority (15/24) were positive by both culture and Xpert Ultra and/or had multiple sputum samples positive (**Table S8**). Of these, 13/15 participants were IGRA positive at Day 1, and 12 of the 13 remained positive at suspected TB visit; one participant had indeterminate IGRA status at the suspected TB visit (**Table S8**). The two participants with negative IGRA status at Day 1 converted to positive at the suspected TB visit. Twelve of the 15 participants were referred for TB treatment and three were not treated. Three of the 15 participants were PLHIV and included one participant from Kenya and two from South Africa (**Table S8**). All three had positive HIV status both at baseline and the suspected TB visit.

Nine (37.5%) of 24 participants with laboratory-confirmed pulmonary TB had one positive TB test result and included five participants in South Africa, and one participant each in India, Indonesia, the Gambia and Zambia (**Table S8**). Of these, 8/9 were IGRA negative at Day 1; 6/8 remained negative at the suspected TB visit and two converted to IGRA positive at the suspected TB visit. One of nine participants was positive both at Day 1 and the suspected TB visit. Four of the nine participants were treated for TB and five were not treated. Two of the nine participants were PLHIV and the remaining seven had negative HIV status (**Table S8**). The PLHIV were from South Africa including one participant with HIV positive status at baseline and one being diagnosed with HIV during the study.

Discussion

In a recent phase 2 TB vaccine trial in South Africa that included 200 PLHIV in the placebo arm, no instances of laboratory confirmed TB were identified (as defined by a single sputum collection for PCR) over a 13-month trial period [1]. In this epidemiologic study, 154 PLHIV in South Africa were enrolled, and at the end of the study, four PLHIV had laboratory-confirmed TB including one participant who was diagnosed with HIV during the study. There were several differences between the two studies, but we speculate that the participants in the phase 2 trial in PLHIV in South Africa may have been protected from TB by stringent eligibility criteria, which included well-controlled antiretroviral therapy and previous completion of TPT, while only <1% of overall participants in this epidemiologic study were on TPT.

References

1. Dagnew AF, Han LL, Naidoo K, et al. Safety and immunogenicity of investigational tuberculosis vaccine M72/AS01E-4 in people living with HIV in South Africa: an observer-blinded, randomised, controlled, phase 2 trial. *The Lancet HIV* 2025.

Tables

Table S2: Disposition by Country

Country	Number of Sites	Screened Set N (%)	All Enrolled Set N (%)	Per-Protocol Set N (%)	Early Discontinuation from Enrolled Set N (%)
Bangladesh	1	160 (100)	160 (100)	160 (100)	12 (7.5)
Brazil	2	322 (100)	319 (99.1)	319 (99.1)	10 (3.1)
Democratic Republic of Congo	3	478 (100)	478 (100)	471 (98.5)	84 (17.6)
The Gambia	1	162 (100)	160 (98.8)	159 (98.1)	21 (13.1)
India	1	160 (100)	160 (100)	160 (100)	0
Indonesia	3	488 (100)	480 (98.4)	480 (98.4)	14 (2.9)
Kenya	4	653 (100)	640 (98.0)	639 (97.9)	51 (8.0)
Mozambique	2	320 (100)	319 (99.7)	319 (99.7)	17 (5.3)
Peru	2	323 (100)	316 (97.8)	316 (97.8)	33 (10.4)
Philippines	7	1121 (100)	1117 (99.6)	1117 (99.6)	25 (2.2)
South Africa	9	1474 (100)	1428 (96.9)	1410 (95.7)	79 (5.5)
Uganda	2	325 (100)	320 (98.5)	320 (98.5)	12 (3.8)
Vietnam	4	636 (100)	627 (98.6)	626 (98.4)	9 (1.4)
Zambia	4	658 (100)	640 (97.3)	639 (97.1)	33 (5.2)

Denominator for percentages is based on the number of participants in Screened Set. Screened Set included all participants for whom the required written informed consent/assent was provided. All enrolled set included all participants who were screened and fulfilled eligibility requirements. Per-protocol set included all participants who fulfilled eligibility requirements and had an IGRA test result for Day 1 visit.

Table S3: Demographics and baseline characteristics by site

Country	Site No.	N	Mean age, years (SD)	25 to 34 years old, n (%)	Females, n (%)	Health-care Profession, n (%)	Mean BMI, kg/m² (SD)	Underweight (<18.5 kg/m²), n (%)	Obese (≥30 kg/m²), n (%)	BCG Scar Present, n (%)	Positive HIV status, n (%)	Ethnicity (Not Hispanic or Latino), n (%)	Race, n (%)					
													Black	Asian	White	South American Indian	South African Coloured	Mixed
Bangladesh	2002	160	25.31 (4.67)	85 (53.1)	68 (42.5)	0	22.60 (4.19)	24 (15.0)	9 (5.6)	158 (98.8)	0	160 (100)	0	156 (97.5)	0	3 (1.9)	1 (0.6)	0
Brazil	1401	159	25.33 (3.92)	85 (53.5)	94 (59.1)	43 (27.0)	26.85 (5.92)	0	33 (20.8)	150 (94.3)	4 (2.5)	0	30 (18.9)	0	117 (73.6)	0	0	12 (7.5)
	1403	160	24.93 (4.44)	87 (54.4)	110 (68.8)	6 (3.8)	27.50 (7.13)	10 (6.3)	53 (33.1)	156 (97.5)	4 (2.5)	160 (100)	87 (54.4)	0	31 (19.4)	0	0	41 (25.6)
Democratic Republic of Congo	2301	155	23.71 (5.47)	62 (40.0)	70 (45.2)	4 (2.6)	21.73 (3.82)	22 (14.2)	2 (1.3)	123 (79.4)	0	155(100)	155 (100)	0	0	0	0	0
	2302	158	21.73 (4.39)	39 (24.7)	65 (41.1)	4 (2.5)	21.33 (2.88)	22 (13.9)	3 (1.9)	146 (92.4)	0	158 (100)	158 (100)	0	0	0	0	0
	2303	158	25.04 (4.93)	79 (50.0)	58 (36.7)	27 (17.1)	21.00 (3.31)	33 (20.9)	4 (2.5)	151 (95.6)	0	158 (100)	158 (100)	0	0	0	0	0
The Gambia	1101	159	23.01 (5.71)	64 (40.3)	109 (68.6)	1 (0.6)	21.75 (4.32)	34 (21.4)	10 (6.3)	103 (64.8)	3 (1.9)	159 (100)	159 (100)	0	0	0	0	0
India	2101	160	25.41 (4.87)	89 (55.6)	50 (31.3)	0	22.84 (2.36)	1 (0.6)	0	159 (99.4)	1 (0.6)	159 (99.4)	0	160 (100)	0	0	0	0
Indonesia	2201	160	22.11 (4.34)	41 (25.6)	60 (37.5)	0	23.32 (5.22)	24 (15.0)	18 (11.3)	79 (49.4)	0	160 (100)	0	160 (100)	0	0	0	0
	2202	160	24.37 (4.77)	70 (43.8)	69 (43.1)	6 (3.8)	23.41 (6.09)	37 (23.1)	23 (14.4)	153 (95.6)	1 (0.6)	160 (100)	0	160 (100)	0	0	0	0
	2203	160	24.26 (4.42)	70 (43.8)	72 (45.0)	0	24.34 (6.58)	30 (18.8)	32 (20.0)	159 (99.4)	0	160 (100)	0	160 (100)	0	0	0	0
Kenya	1201	160	23.83 (4.60)	61 (38.1)	91 (56.9)	6 (3.8)	22.66 (4.44)	20 (12.5)	14 (8.8)	149 (93.1)	5 (3.1)	160 (100)	160 (100)	0	0	0	0	0
	1202	160	25.45 (4.62)	87 (54.4)	83 (51.9)	1 (0.6)	24.93 (6.20)	9 (5.6)	31 (19.4)	153 (95.6)	9 (5.6)	159 (99.4)	160 (100)	0	0	0	0	0
	1203	160	26.69 (4.10)	115 (71.9)	100 (62.5)	1 (0.6)	24.08 (4.38)	3 (1.9)	20 (12.5)	158 (98.8)	24 (15.0)	159 (99.4)	160 (100)	0	0	0	0	0

Country	Site No.	N	Mean age, years (SD)	25 to 34 years old, n (%)	Females, n (%)	Health-care Profession, n (%)	Mean BMI, kg/m² (SD)	Underweight (<18.5 kg/m²), n (%)	Obese (≥30 kg/m²), n (%)	BCG Scar Present, n (%)	Positive HIV status, n (%)	Ethnicity (Not Hispanic or Latino), n (%)	Race, n (%)					
													Black	Asian	White	South American Indian	South African Coloured	Mixed
	1204	159	24.79 (4.38)	74 (46.5)	68 (42.8)	2 (1.3)	22.75 (5.08)	16 (10.1)	16 (10.1)	139 (87.4)	2 (1.3)	159 (100)	159 (100)	0	0	0	0	0
Mozambique	1601	160	21.84 (5.10)	46 (28.8)	90 (56.3)	0	22.62 (3.94)	17 (10.6)	6 (3.8)	159 (99.4)	27 (16.9)	159 (99.4)	160 (100)	0	0	0	0	0
	1602	159	22.14 (5.05)	45 (28.3)	68 (42.8)	1 (0.6)	22.04 (3.77)	13 (8.2)	10 (6.3)	149 (93.7)	2 (1.3)	158 (99.4)	159 (100)	0	0	0	0	0
Peru	1701	156	23.77 (4.33)	56 (35.9)	88 (56.4)	12 (7.7)	26.96 (5.10)	4 (2.6)	35 (22.4)	114 (73.1)	1 (0.6)	0	0		2 (1.3)	5 (3.2)	0	148 (94.9)
	1702	160	26.20 (4.50)	100 (62.5)	100 (62.5)	0	27.87 (5.98)	1 (0.6)	49 (30.6)	135 (84.4)	2 (1.3)	0	0	0	0	0	0	160 (100)
Philippines	1501	160	24.46 (5.05)	75 (46.9)	87 (54.4)	0	23.68 (4.89)	22 (13.8)	10 (6.3)	114 (71.3)	0	160 (100)	0	160 (100)	0	0	0	0
	1502	160	23.89 (5.40)	68 (42.5)	95 (59.4)	7 (4.4)	25.31 (5.49)	16 (10.0)	30 (18.8)	119 (74.4)	0	160 (100)	0	160 (100)	0	0	0	0
	1503	158	25.18 (4.94)	80 (50.6)	85 (53.8)	0	24.18 (4.64)	14 (8.9)	17 (10.8)	122 (77.2)	1 (0.6)	158 (100)	0	158 (100)	0	0	0	0
	1504	160	23.89 (4.74)	63 (39.4)	68 (42.5)	8 (5.0)	23.78 (4.91)	21 (13.1)	17 (10.6)	159 (99.4)	2 (1.3)	159 (99.4)	0	160 (100)	0	0	0	0
	1505	159	24.31 (5.13)	71 (44.7)	77 (48.4)	3 (1.9)	23.79 (4.72)	11 (6.9)	18 (11.3)	108 (67.9)	0	159 (100)	0	159 (100)	0	0	0	0
	1507	160	24.94 (4.53)	79 (49.4)	82 (51.3)	0	25.50 (4.94)	5 (3.1)	25 (15.6)	131 (81.9)	1 (0.6)	160 (100)	0	160 (100)	0	0	0	0
	1508	160	24.94 (4.50)	77 (48.1)	57 (35.6)	0	26.16 (5.15)	4 (2.5)	39 (24.4)	116 (72.5)	2 (1.3)	160 (100)	0	160 (100)	0	0	0	0
South Africa	1001	158	25.13 (4.73)	83 (52.5)	87 (55.1)	4 (2.5)	25.29 (6.97)	11 (7.0)	36 (22.8)	129 (81.6)	0	154 (97.5)	158 (100)	0	0	0	0	0
	1002	154	24.71 (5.83)	82 (53.2)	91 (59.1)	0	26.17 (7.48)	10 (6.5)	40 (26.0)	68 (44.2)	30 (19.5)	154 (100)	154 (100)	0	0	0	0	0
	1003	160	24.66 (4.55)	80 (50.0)	122 (76.3)	1 (0.6)	27.45 (7.90)	8 (5.0)	53 (33.1)	86 (53.8)	28 (17.5)	160 (100)	160 (100)	0	0	0	0	0
	1004	158	23.72 (4.20)	61 (38.6)	58 (36.7)	4 (2.5)	21.92 (5.23)	46 (29.1)	11 (7.0)	92 (58.2)	3 (1.9)	158 (100)	66 (41.8)	0	1 (0.6)	0	90 (57.0)	1 (0.6)

Country	Site No.	N	Mean age, years (SD)	25 to 34 years old, n (%)	Females, n (%)	Health-care Profession, n (%)	Mean BMI, kg/m^2 (SD)	Under weight (<18.5 kg/m^2), n (%)	Obese (≥30 kg/m^2), n (%)	BCG Scar Present, n (%)	Positive HIV status, n (%)	Ethnicity (Not Hispanic or Latino), n (%)	Race, n (%)					
													Black	Asian	White	South American Indian	South African Coloured	Mixed
	1006	155	25.92 (4.72)	91 (58.7)	87 (56.1)	1 (0.6)	23.86 (6.91)	30 (19.4)	27 (17.4)	83 (53.5)	30 (19.4)	155 (100)	123 (79.4)	0	0	0	32 (20.6)	0
	1007	160	23.47 (5.14)	66 (41.3)	72 (45.0)	0	21.72 (5.49)	57 (35.6)	15 (9.4)	75 (46.9)	10 (6.3)	160 (100)	140 (87.5)	0	0	0	20 (12.5)	0
	1008	147	24.19 (5.08)	63 (42.9)	69 (46.9)	0	23.06 (6.45)	43 (29.3)	23 (15.6)	87 (59.2)	12 (8.2)	147 (100)	135 (91.8)	0	12 (8.2)	0	0	0
	1009	158	24.23 (4.26)	70 (44.3)	54 (34.2)	0	22.51 (5.40)	32 (20.3)	16 (10.1)	105 (66.5)	10 (6.3)	158 (100)	158 (100)	0	0	0	0	0
	1010	160	25.18 (4.74)	88 (55.0)	126 (78.8)	0	27.91 (6.97)	7 (4.4)	59 (36.9)	68 (42.5)	31 (19.4)	151 (94.4)	160 (100)	0	0	0	0	0
Uganda	1801	160	24.67 (4.69)	76 (47.5)	82 (51.3)	7 (4.4)	23.31 (4.75)	11 (6.9)	16 (10.0)	128 (80.0)	12 (7.5)	160 (100)	160 (100)	0	0	0	0	0
	1802	160	24.23 (3.95)	71 (44.4)	79 (49.4)	2 (1.3)	23.03 (4.24)	15 (9.4)	11 (6.9)	114 (71.3)	8 (5.0)	159 (99.4)	160 (100)	0	0	0	0	0
Vietnam	1901	154	24.69 (5.13)	75 (48.7)	77 (50.0)	26 (16.9)	23.28 (4.62)	21 (13.6)	16 (10.4)	132 (85.7)	3 (1.9)	154 (100)	0	154 (100)	0	0	0	0
	1902	155	22.88 (4.69)	44 (28.4)	58 (37.4)	1 (0.6)	23.72 (5.02)	15 (9.7)	20 (12.9)	153 (98.7)	2 (1.3)	155 (100)	0	155 (100)	0	0	0	0
	1903	160	23.32 (3.34)	49 (30.6)	103 (64.4)	48 (30.0)	21.17 (3.23)	33 (20.6)	4 (2.5)	141 (88.1)	0	160 (100)	0	160 (100)	0	0	0	0
	1904	157	23.75 (5.79)	71 (45.2)	84 (53.5)	3 (1.9)	22.23 (4.31)	30 (19.1)	10 (6.4)	138 (87.9)	0	157 (100)	0	157 (100)	0	0	0	0
Zambia	1301	160	22.84 (4.26)	57 (35.6)	102 (63.8)	4 (2.5)	22.13 (3.93)	17 (10.6)	9 (5.6)	108 (67.5)	16 (10.0)	160 (100)	160 (100)	0	0	0	0	0
	1302	159	22.84 (5.15)	63 (39.6)	123 (77.4)	3 (1.9)	22.26 (4.60)	23 (14.5)	11 (6.9)	103 (64.8)	14 (8.8)	159 (100)	159 (100)	0	0	0	0	0
	1303	160	23.85 (5.14)	71 (44.4)	72 (45.0)	10 (6.3)	22.72 (4.34)	13 (8.1)	12 (7.5)	118 (73.8)	18 (11.3)	160 (100)	160 (100)	0	0	0	0	0
	1304	160	23.76 (4.35)	62 (38.8)	121 (75.6)	3 (1.9)	21.06 (3.80)	33 (20.6)	4 (2.5)	97 (60.6)	18 (11.3)	160 (100)	160 (100)	0	0	0	0	0

Denominator for percentages is based on the number of participants in the per-protocol set. BMI=Body Mass Index derived as weight (kg)/height(m^2). One participant each had ‘other’ ethnicity at site 1403 in Brazil, site 1701 in Peru.

Table S4: IGRA Status at Day 1 by site (per-protocol set)

Country	Site No.	N1[1]	Positive, n (% [95% CI])	Negative, n (%)	Indeterminate, n (%)
Bangladesh	2002	160	70 (43.8 [35.8–51.7])	88 (55.0)	2 (1.3)
Brazil	1401	159	11 (6.9 [2.7–11.2])	148 (93.1)	0
	1403	160	60 (37.5 [29.7–45.3])	100 (62.5)	0
Democratic Republic of Congo	2301	155	53 (34.2 [26.4–42.0])	94 (60.6)	8 (5.2)
	2302	158	77 (48.7 [40.6–56.8])	68 (43.0)	13 (8.2)
	2303	158	79 (50.0 [41.9–58.1])	75 (47.5)	4 (2.5)
The Gambia	1101	159	37 (23.3 [16.4–30.2])	119 (74.8)	3 (1.9)
India	2101	160	3 (1.9 [0–4.3])	152 (95.0)	5 (3.1)
Indonesia	2201	160	43 (26.9 [19.7–34.1])	117 (73.1)	0
	2202	160	59 (36.9 [29.1–44.7])	100 (62.5)	1 (0.6)
	2203	160	59 (36.9 [29.1–44.7])	100 (62.5)	1 (0.6)
Kenya	1201	160	57 (35.6 [27.9–43.4])	102 (63.8)	1 (0.6)
	1202	160	75 (46.9 [38.8–54.9])	82 (51.3)	3 (1.9)
	1203	160	56 (35.0 [27.3–42.7])	100 (62.5)	4 (2.5)
	1204	159	62 (39.0 [31.1–46.9])	96 (60.4)	1 (0.6)
Mozambique	1601	160	21 (13.1 [7.6–18.7])	137 (85.6)	2 (1.3)
	1602	159	25 (15.7 [9.8–21.7])	128 (80.5)	6 (3.8)
Peru	1701	156	33 (21.2 [14.4–27.9])	119 (76.3)	4 (2.6)
	1702	160	33 (20.6 [14.0–27.2])	126 (78.8)	1 (0.6)
Philippines	1501	160	59 (36.9 [29.1–44.7])	101 (63.1)	0
	1502	160	17 (10.6 [5.5–15.7])	141 (88.1)	2 (1.3)
	1503	158	61 (38.6 [30.7–46.5])	82 (51.9)	15 (9.5)
	1504	160	16 (10.0 [5.0–15.0])	144 (90.0)	0
	1505	159	29 (18.2 [11.9–24.6])	130 (81.8)	0
	1507	160	30 (18.8 [12.4–25.1])	129 (80.6)	1 (0.6)
	1508	160	27 (16.9 [10.8–23.0])	133 (83.1)	0

Country	Site No.	N1[1]	Positive, n (% [95% CI])	Negative, n (%)	Indeterminate, n (%)
South Africa	1001	158	63 (39.9 [31.9–47.8])	95 (60.1)	0
	1002	154	41 (26.6 [19.3–33.9])	112 (72.7)	1 (0.6)
	1003	160	60 (37.5 [29.7–45.3])	95 (59.4)	5 (3.1)
	1004	158	79 (50.0 [41.9–58.1])	79 (50.0)	0
	1006	155	91 (58.7 [50.6–66.8])	64 (41.3)	0
	1007	160	83 (51.9 [43.8–59.9])	75 (46.9)	2 (1.3)
	1008	147	53 (36.1 [28.0–44.2])	94 (63.9)	0
	1009	158	42 (26.6 [19.4–33.8])	113 (71.5)	3 (1.9)
	1010	160	85 (53.1 [45.1–61.2])	71 (44.4)	4 (2.5)
Uganda	1801	160	51 (31.9 [24.3–39.4])	99 (61.9)	10 (6.3)
	1802	160	58 (36.3 [28.5–44.0])	96 (60.0)	6 (3.8)
Vietnam	1901	154	16 (10.4 [5.2–15.5])	134 (87.0)	4 (2.6)
	1902	155	18 (11.6 [6.2–17.0])	137 (88.4)	0
	1903	160	7 (4.4 [0.9–7.9])	150 (93.8)	3 (1.9)
	1904	157	31 (19.7 [13.2–26.3])	124 (79.0)	2 (1.3)
Zambia	1301	160	74 (46.3 [38.2–54.3])	84 (52.5)	2 (1.3)
	1302	159	78 (49.1 [41.0–57.1])	78 (49.1)	3 (1.9)
	1303	160	24 (15.0 [9.2–20.8])	89 (55.6)	47(29.4)
	1304	160	62 (38.8 [30.9–46.6])	90 (56.3)	8 (5.0)

[1]N1 is the number of participants in the per-protocol set and the percentages for IGRA status at screening are based on N1. 95% CI was presented based on binomial distribution assumption, using the Wald method with continuity correction. IGRA=Interferon-gamma release assay

Table S5: IGRA Status at Day 1 by Age Category (per-protocol set)

		15-24 years old				25-34 years old			
**Country**	**Site No.**	**N**	**Positive, n (% [95%CI])**	**Negative, n (%)**	**Indt, n (%)**	**N**	**Positive, n (% [95%CI])**	**Negative, n (%)**	**Indt, n (%)**
Bangladesh	2002	75	32 (42.7 [31.3–54.6]	43 (57.3)	0	85	38 (44.7 [33.9–55.9])	45 (52.9)	2 (2.4)
Brazil	1401	74	2 (2.7 [0.3–9.4]	72 (97.3)	0	85	9 (10.6 [5.0–19.2])	76 (89.4)	0
	1403	73	27 (37.0 [26.0–49.1])	46 (63.0)	0	87	33 (37.9 [27.7–49.0])	54 (62.1)	0
Democratic Republic of Congo	2301	93	29 (31.2 [22.0–41.6])	60 (64.5)	4 (4.3)	62	24 (38.7 [26.6–51.9])	34 (54.8)	4 (6.5)
	2302	119	58 (48.7 [39.3–58.1])	54 (45.4)	7 (5.9)	39	19 (48.7 [32.4–65.2])	14 (35.9)	6 (15.4)
	2303	79	32 (40.5 [29.6–52.1])	44 (55.7)	3 (3.8)	79	47 (59.5[(47.9–70.4])	31 (39.2)	1 (1.3)
The Gambia	1101	95	16 (16.8 [9.9–25.9])	79 (83.2)	0	64	21 (32.8 [21.6–45.7])	40 (62.5)	3 (4.7)
India	2101	71	0 (0 [0–5.1])	69 (97.2)	2 (2.8)	89	3 (3.4 [0.7–9.5])	83 (93.3)	3 (3.4)
Indonesia	2201	119	24 (20.2 [12.5–27.8])	95 (79.8)	0	41	19 (46.3 [30.7–62.6])	22 (53.7)	0
	2202	90	31 (34.4 [24.7–45.2])	58 (64.4)	1 (1.1)	70	28 (40.0 [28.5–52.4])	42 (60.0)	0
	2203	90	29 (32.2 [22.8-42.9])	60 (66.7)	1 (1.1)	70	30 (42.9 [31.1–55.3])	40 (57.1)	0
Kenya	1201	99	34 (34.3 [25.1–44.6])	65 (65.7)	0	61	23 (37.7 [25.6–51.0])	37 (60.7)	1 (1.6)
	1202	73	27 (37.0 [26.0–49.1])	46 (63.0)	0	87	48 (55.2 [44.1–65.9])	36 (41.4)	3 (3.4)
	1203	45	14 (31.1 [18.2–46.6])	31 (68.9)	0	115	42 (36.5 [27.3–45.8])	69 (60.0)	4 (3.5)
	1204	85	33 (38.8 [28.4–50.0])	52 (61.2)	0	74	29 (39.2 [28.0–51.2])	44 (59.5)	1 (1.4)
Mozambique	1601	114	15 (13.2 [6.5–19.8])	98 (86.0)	1 (0.9)	46	6 (13.0 [4.9–26.3])	39 (84.8)	1 (2.2)
	1602	114	18 (15.8 [8.7–22.9])	91 (79.8)	5 (4.4)	45	7 (15.6 [6.5–29.5])	37 (82.2)	1 (2.2)
Peru	1701	100	15 (15.0 [7.5–22.5])	82 (82.0)	3 (3.0)	56	18 (32.1 [20.3–46.0])	37 (66.1)	1 (1.8)
	1702	60	12 (20.0 [10.8–32.3])	48 (80.0)	0	100	21 (21.0 [12.5–29.5])	78 (78.0)	1 (1.0)
Philippines	1501	85	27 (31.8 [22.1–42.8])	58 (68.2)	0	75	32 (42.7 [31.3–54.6])	43 (57.3)	0
	1502	92	8 (8.7 [3.8–16.4])	82 (89.1)	2 (2.2)	68	9 (13.2 [6.2–23.6])	59 (86.8)	0
	1503	78	31 (39.7 [28.8–51.5])	42 (53.8)	5 (6.4)	80	30 (37.5 [26.9–49.0])	40 (50.0)	10 (12.5)
	1504	97	5 (5.2 [1.7–11.6])	92 (94.8)	0	63	11 (17.5 [9.1–29.1])	52 (82.5)	0
	1505	88	13 (14.8 [8.1–23.9])	75 (85.2)	0	71	16 (22.5 [13.5–34.0])	55 (77.5)	0
	1507	81	11 (13.6 [7.0–23.0])	70 (86.4)	0	79	19 (24.1 [15.1–35.0])	59 (74.7)	1 (1.3)
	1508	83	13 (15.7 [8.6–25.3])	70 (84.3)	0	77	14 (18.2 [10.3–28.6])	63 (81.8)	0
South Africa	1001	75	25 (33.3 [22.9–45.2])	50 (66.7)	0	83	38 (45.8 [34.8–57.1])	45 (54.2)	0

		15-24 years old				25-34 years old			
**Country**	**Site No.**	**N**	**Positive, n (% [95%CI])**	**Negative, n (%)**	**Indt, n (%)**	**N**	**Positive, n (% [95%CI])**	**Negative, n (%)**	**Indt, n (%)**
	1002	72	12 (16.7 [8.9–27.3])	60 (83.3)	0	82	29 (35.4 [25.1–46.7])	52 (63.4)	1 (1.2)
	1003	80	23 (28.8 [19.2–40.0])	54 (67.5)	3 (3.8)	80	37 (46.3 [35.0–57.8])	41 (51.3)	2 (2.5)
	1004	97	46 (47.4 [37.2–57.8])	51 (52.6)	0	61	33 (54.1 [40.8–66.9])	28 (45.9)	0
	1006	64	37 (57.8 [44.8–70.1])	27 (42.2)	0	91	54 (59.3 [48.5–69.5])	37 (40.7)	0
	1007	94	41 (43.6 [33.4–54.2])	52 (55.3)	1 (1.1)	66	42 (63.6 [50.9–75.1])	23 (34.8)	1 (1.5)
	1008	84	24 (28.6 [19.2–39.5])	60 (71.4)	0	63	29 (46.0 [33.4–59.1])	34 (54.0)	0
	1009	88	21 (23.9 [15.4–34.1])	66 (75.0)	1 (1.1)	70	21 (30.0 [19.6–42.1])	47 (67.1)	2 (2.9)
	1010	72	28 (38.9 [27.6–51.1])	41 (56.9)	3 (4.2)	88	57 (64.8 [53.9–74.7])	30 (34.1)	1 (1.1)
Uganda	1801	84	23 (27.4 [18.2–38.2])	56 (66.7)	5 (6.0)	76	28 (36.8 [26.1–48.7])	43 (56.6)	5 (6.6)
	1802	89	27 (30.3 [21.0–41.0])	60 (67.4)	2 (2.2)	71	31 (43.7 [31.9–56.0])	36 (50.7)	4 (5.6)
Vietnam	1901	79	7 (9.0 [3.6–17.4])	70 (88.6)	2 (2.5)	75	9 (12.0 [5.6–21.6])	64 (85.3)	2 (2.7)
	1902	111	10 (9.0 [3.2–14.8])	101 (91.0)	0	44	8 (18.2 [8.2–32.7])	36 (81.8)	0
	1903	111	4 (3.6 [0–7.5])	104 (93.7)	3 (2.7)	49	3 (6.1 [1.3–16.9])	46 (93.9)	0
	1904	86	17 (19.8 [12.0–29.8])	68 (79.1)	1 (1.2)	71	14 (19.7 [11.2–30.9])	56 (78.9)	1 (1.4)
Zambia	1301	103	44 (42.7 [32.7–52.8])	57 (55.3)	2 (1.9)	57	30 (52.6 [39.0–66.0])	27 (47.4)	0
	1302	96	44 (45.8 [35.6–56.3])	50 (52.1)	2 (2.1)	63	34 (54.0 [40.9–66.6])	28 (44.4)	1 (1.6)
	1303	89	13 (14.6 [8.0–23.7])	46 (51.7)	30 (33.7)	71	11 (15.5 [8.0–26.0])	43 (60.6)	17 (23.9)
	1304	98	32 (32.7 [23.5–42.9])	59 (60.2)	7 (7.1)	62	30 (48.4 [35.5–61.4])	31 (50.0)	1 (1.6)

Denominator for percentages is the total number of participants with an IGRA status in the specified age group. 95% CI was presented based on binomial distribution assumption, using the Wald method with continuity correction if the sample size was 100 or greater, and Clopper-Pearson method for sample size less than 100, utilizing site level sample size per age category. IGRA=Interferon-gamma release assay, Indt = indeterminate

Table S6: Change in IGRA status from Day 1 to Month 12, by site (per-protocol set)

Country	Site Number	Participants with IGRA Status at M12, N	Participants with IGRA Reversion (D1 Positive and M12 Negative), n (% [95%CI])	Participants with IGRA Conversion (D1 Negative and M12 Positive), n (% [95%CI])
Bangladesh	2002	144	13 (9.0 [4.0–14.1])	24 (16.7 [10.2–23.1])
	1401	120	4 (3.3 [0–7.0])	4 (3.3 [0–7.0])
Brazil	1403	157	0	15 (9.6 [4.6–14.5])
Democratic Republic of Congo	2301	100	5 (5.0 [0.2–9.8])	14 (14.0 [6.7–21.3])
	2302	108	6 (5.6 [0.8–10.3])	13 (12.0 [5.4–18.6])
	2303	126	6 (4.8 [0.6–8.9])	12 (9.5 [4.0–15.0])
The Gambia	1101	124	7 (5.6 [1.2–10.1])	8 (6.5 [1.7–11.2])
India	2101	130	1 (0.8 [0–2.7])	2 (1.5 [0–4.0])
Indonesia	2201	160	2 (1.3 [0–3.3])	19 (11.9 [6.5–17.2])
	2202	152	8 (5.3 [1.4–9.1])	8 (5.3 [1.4–9.1])
	2203	152	3 (2.0 [0–4.5])	16 (10.5 [5.3–15.7])
Kenya	1201	151	18 (11.9 [6.4–17.4])	7 (4.6 [1.0–8.3])
	1202	126	7 (5.6 [1.2–10.0])	7 (5.6 [1.2–10.0])
	1203	130	5 (3.8 [0.2–7.5])	6 (4.6 [0.6–8.6])
	1204	132	4 (3.0 [0–6.3])	4 (3.0 [0–6.3])
Mozambique	1601	139	3 (2.2 [0–4.9])	3 (2.2 [0–4.9])
	1602	110	7 (6.4 [1.3–11.4])	3 (2.7 [0–6.2])
Peru	1701	127	3 (2.4 [0–5.4])	6 (4.7 [0.6–8.8])
	1702	143	9 (6.3 [2.0–10.6])	3 (2.1 [0–4.8])
Philippines	1501	158	14 (8.9 [4.1–13.6])	18 (11.4 [6.1–16.7])
	1502	152	2 (1.3 [0–3.5])	28 (18.4 [11.9–24.9])
	1503	131	14 (10.7 [5.0–16.4])	16 (12.2 [6.2–18.2])
	1504	153	4 (2.6 [0–5.5])	10 (6.5 [2.3–10.8])
	1505	154	10 (6.5 [2.3–10.7])	13 (8.4 [3.7–13.2])
	1507	158	0	51 (32.3 [24.7–39.9])
	1508	155	8 (5.2 [1.4–9.0])	11 (7.1 [2.7–11.5])
South Africa	1001	149	4 (2.7 [0–5.6])	7 (4.7 [1.0–8.4])
	1002	144	5 (3.5 [0.1–6.8])	4 (2.8 [0–5.8])
	1003	134	7 (5.2 [1.1–9.4])	7 (5.2 [1.1–9.4])
	1004	148	3 (2.0 [0–4.6])	7 (4.7 [1.0–8.5])
	1006	145	3 (2.1 [0–4.7])	7 (4.8 [1.0–8.7])
	1007	142	10 (7.0 [2.5–11.6])	6 (4.2 [0.6–7.9])
	1008	133	3 (2.3 [0–5.2])	5 (3.8 [0.2–7.4])
	1009	141	1 (0.7 [0–2.4])	7 (5.0 [1.0–8.9])
	1010	152	5 (3.3 [0.1–6.5])	8 (5.3 [1.4–9.1])
Uganda	1801	136	2 (1.5 [0–3.9])	7 (5.1 [1.1–9.2])
	1802	137	8 (5.8 [1.5–10.1])	9 (6.6 [2.1–11.1])
Vietnam	1901	141	3 (2.1 [0–4.9])	21 (14.9 [8.7–21.1])
	1902	154	5 (3.2 [0.1–6.4])	3 (1.9 [0.0–4.5])
	1903	150	0	6 (4.0 [0.5–7.5])
	1904	149	6 (4.0 [0.5–7.5])	8 (5.4 [1.4–9.3])
Zambia	1301	114	8 (7.0 [1.9–12.1])	4 (3.5 [0–7.3])

Country	Site Number	Participants with IGRA Status at M12, N	Participants with IGRA Reversion (D1 Positive and M12 Negative), n (% [95%CI])	Participants with IGRA Conversion (D1 Negative and M12 Positive), n (% [95%CI])
	1302	123	4 (3.3 [0–6.8])	10 (8.1 [2.9–13.4])
	1303	98	3 (3.1 [0–7.0])	30 (30.6 [21.0–40.2])
	1304	117	7 (6.0 [1.3–10.7])	9 (7.7 [2.4–12.9])

Denominator (N) for percentages is the number of participants with IGRA status positive and negative at month 12. IGRA=Interferon-gamma release assay; D1=Day 1; M12=Month 12

Table S7: Incidence rate of suspected pulmonary TB by country (per-protocol set)

Country	Number of participants in per-protocol set	Number of participants with first suspected TB	Person Years	Incidence Rate [IR/100,000]	95% CI for IR
Brazil	319	18	391.6	4596.6	2751.8–7145.3
Democratic Republic of Congo	471	4	669.7	597.2	163.1–1519.1
The Gambia	159	2	254.0	787.5	95.6–2798.4
India	160	3	161.6	1856.7	384.6–5329.0
Indonesia	480	10	435.3	2297.5	1106.6–4188.2
Kenya	639	16	958.9	1668.7	958.2–2688.6
Mozambique	319	3	338.8	885.4	183.0–2564.1
Peru	316	11	441.8	2490.1	1252.0–4394.3
Philippines	1117	31	1688.1	1836.4	1252.8–2591.4
South Africa	1410	260	2089.7	12441.7	11097.5–13875.3
Uganda	320	17	453.8	3745.8	2203.3–5901.5
Vietnam	626	11	889.7	1236.4	619.4–2196.9
Zambia	639	24	875.4	2741.5	1767.2–4041.7

Bangladesh had no cases of suspected TB. The Suspected Pulmonary TB incidence rates were calculated as the number of first suspected Pulmonary TB event presented during the study follow-up period, divided by the number of participant days at risk during the follow-up period and expressed as the number of events per 100,000 participant-years. The corresponding 95% 2-sided confidence interval associated with the incidence rate of Suspected Pulmonary TB overall were derived based on binomial distribution assumption. CI=Confidence interval; IR=Incidence rate; TB=Tuberculosis

Table S8: IGRA status, HIV status and TB treatment of participants with laboratory-confirmed pulmonary TB

Countries (n)	Direct Mycobacterial Detection		IGRA status		HIV status		Referred for TB treatment (yes/no)
Laboratory-confirmed TB cases with multiple positive TB test results							
	MGIT™	Xpert® MTB/RIF Ultra	Day 1	Suspected TB visit	Day 1	Suspected TB visit	
Indonesia (1)	-	+	+	+	-	-	yes
Kenya (3)	+	+	+	+	-	-	yes
	+	+	+	+	+	+	yes
	+	+	+	+	-	-	yes
Peru (1)	+	+	+	+	-	-	no
Philippines (4)	-	+	+	+	-	-	yes
	+	+	+	+	-	-	yes
	+	+	-	+	-	-	yes
	+	+	+	+	-	-	yes
South Africa (4)	+	+[a]	-	+	+	+	yes
	+	+	+	+	-	-	yes
	+	+	+	+	-	-	no
	+	+	+	+	+	+	no
Vietnam (2)	-	+[a]	+	Indt	-	-	yes
	-	+	+	+	-	-	yes
Laboratory-confirmed TB cases with one positive TB test result							
The Gambia (1)	-	+	-	+	-	Not done	yes
India (1)	Not done	+[a]	-	-	-	-	yes
Indonesia (1)	+	-	-	-	-	-	no
South Africa (5)	-	+	-	-	-	-	no
	+	-	+	+	+	+	yes
	+	-	-	-	-	-	no
	+	-	-	-	-	-	yes
	-	+[a]	-	-	-	+	no
Zambia (1)	-	+	-	+	-	-	no

n=participants with laboratory confirmed pulmonary TB. [a]Trace result from Xpert® MTB/RIF Ultra assay was used in case determination. HIV=Human immunodeficiency virus; IGRA=Interferon-gamma release assay; Indt=indeterminate;TB=Tuberculosis.

Table S9: Incidence rate of clinical TB by country (per-protocol set)

Country	Number of participants in per-protocol set	Number of participants in with first clinical TB	Person Years	Incidence Rate [IR/100,000]	95% CI for IR
Brazil	319	2	404.3	494.6	60.0–1772.2
Indonesia	480	3	438.7	683.8	141.2–1986.4
Kenya	639	3	973.2	308.3	63.6–896.8
Philippines	1117	7	1706.4	410.2	165.2–842.4
South Africa	1410	7	2331.1	300.3	120.9–617.1
Vietnam	626	1	897.3	111.4	2.8–618.7
Zambia	639	8	894.5	894.3	387.2–1751.5

Bangladesh, Democratic Republic of Congo, the Gambia, India, Mozambique, Peru and Uganda had no cases of clinical TB. The clinical TB incidence rates were calculated as the number of first clinical TB event presented during the study follow-up period, divided by the number of participant days at risk during the follow-up period and expressed as the number of events per 100,000 participant-years. The corresponding 95% 2-sided confidence intervals associated with the incidence rate of clinical TB overall were derived based on binomial distribution assumption. CI=Confidence interval; IR=Incidence rate; TB=Tuberculosis

Figure S1: Study Profile. Of the 7,280 participants screened, 116 were excluded due to ineligibility. A total of 7,164 participants were enrolled in the study. Of these, 29 were excluded from the per-protocol population due to missing IGRA result at screening, resulting in a per-protocol set of 7,135 participants. A total of 6,764 participants completed the study and 400 discontinued.

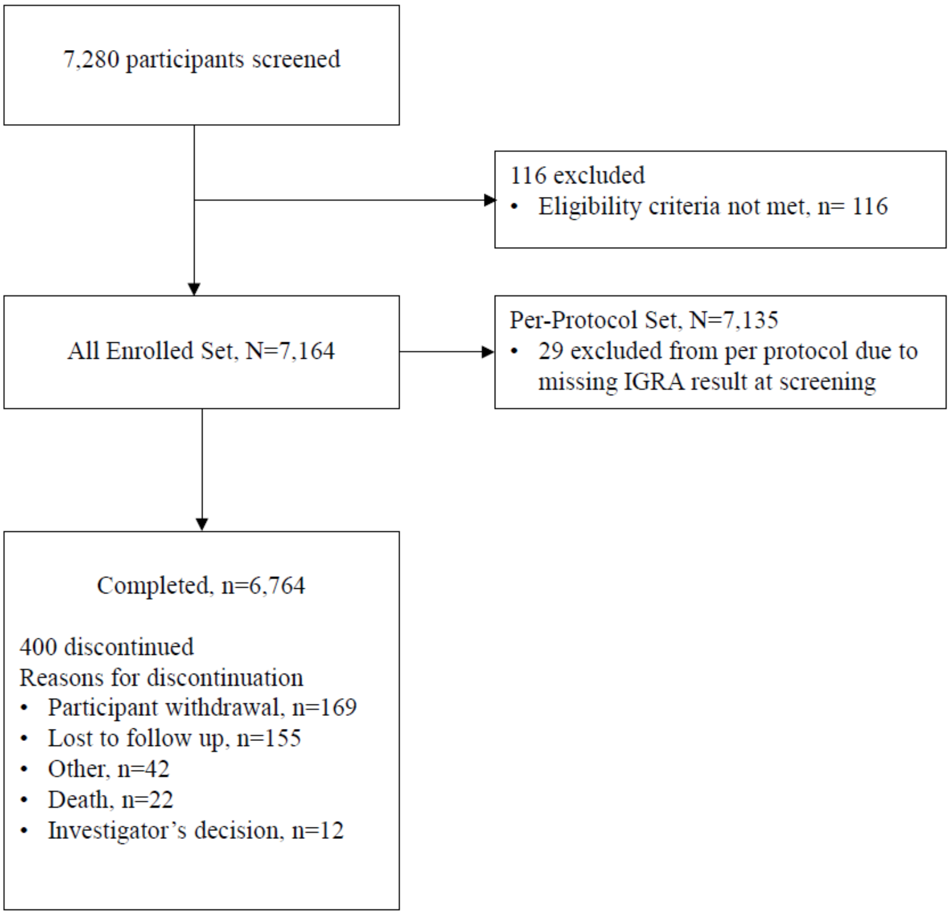

Supplement: Supplementary file 1 [file ijtldopen25-0684_supplementarydata1.pdf]
